# Capturing Collaborative Challenges: Designing Complexity-Sensitive Theories of Change for Cross-Sector Partnerships

**DOI:** 10.1007/s10551-018-3857-7

**Published:** 2018-04-09

**Authors:** Rob van Tulder, Nienke Keen

**Affiliations:** 1 Partnerships Resource Centre, RSM Erasmus University Rotterdam, Burgemeester Oudlaan 50, 3062 PA Rotterdam, The Netherlands; 2 AgriProFocus, Utrecht, The Netherlands

**Keywords:** Theories of Change, Transformative partnerships, Complexity alignment framework

## Abstract

Systems change requires complex interventions. Cross-sector partnerships (CSPs) face the daunting task of addressing complex societal problems by aligning different backgrounds, values, ideas and resources. A major challenge for CSPs is how to link the type of partnership to the intervention needed to drive change. Intervention strategies are thereby increasingly based on Theories of Change (ToCs). Applying ToCs is often a donor requirement, but it also reflects the ambition of a partnership to enhance its transformative potential. The current use of ToCs in partnering efforts varies greatly. There is a tendency for a linear and relatively simple use of ToCs that does limited justice to the complexity of the problems partnerships aim to address. Since partnership dynamics are already complex and challenging themselves, confusion and disagreement over the appropriate application of ToCs is likely to hamper rather than enhance the transformative potential of partnerships. We develop a complexity alignment framework and a diagnostic tool that enables partnerships to better appreciate the complexity of the context in which they operate, allowing them to adjust their learning strategy. This paper applies recent insights into how to deal with complexity from both the evaluation and theory of change fields to studies investigating the transformative capacity of partnerships. This can (1) serve as a check to define the challenges of partnering projects and (2) can help delineate the societal sources and layers of complexity that cross-sector partnerships deal with such as failure, insufficient responsibility taking and collective action problems at four phases of partnering.

## Introduction: Designing Collaborative Interventions for Systemic Change

Systemic change processes are by default ‘complex’, ‘grand’ (Colguitt and George 2011) or even ‘wicked’ (Rittel and Webber 1973; Waddock et al. 2015). Systemic change is usually defined as ‘change that pervades all parts of a system, taking into account the interrelationships and interdependencies among those parts*’.*^1^ Cross-Sector Partnerships (CSPs) are considered to be a viable, needed and constructive approach to address interrelated problems that either originate in the failure of individual organisations and societal sectors (Kolk et al. 2008) and transcend the scope of influence of individual societal sectors (Clarke and Fuller 2010; Selsky and Parker 2005) or represent collective action problems while aiming at innovative approaches (Patton 2011) and the creation of positive systemic effects (Googins and Rochlin 2000). Partnership is one of five universal building blocks of all UN Global Goals, arguably the leading agenda for systemic change at a global scale (UN 2015). CSPs are thus rapidly becoming a dominant approach to systemic change (PrC 2014; Seitanidi and Crane 2014) with almost paradigmatic status (Austin 2000; Glasbergen et al. 2007).

Partnership practice has been criticised for not addressing systemic change adequately, for instance, due to sub-optimal partnering configurations (Wettenhall 2003), a private sector that is too dominant (Dauvergne and LeBaron 2014; Mintzberg 2015), ambitions that are too limited, or issue-partner fits that are not optimal (Van Tulder and Pfisterer 2014). Social systems theory talks about ‘collaborative complexities’ (Schneider et al. 2017). Ill-conceived partnerships can undermine the legitimacy of the whole phenomenon (Bäckstrand 2006), for instance, due to overly optimistic or superficial claims, lacking responsibilities or poor governance structures (Brinkerhoff and Brinkerhoff 2011). There is growing evidence that collaboration does not come easy. Success is hardly assured (Bryson et al. 2015), not in the least because processes of multiple-stakeholder engagement do not address the actual complexity of the problem (Pattberg and Widerberg 2016). The CSP literature acknowledges that complex problems need more complex interventions (Austin and Seitanidi 2012), but falls short of delineating what this implies in the design phase of a partnership. The practical relevance of the idea of ‘collaborative advantage’ (Huxham and Vangen 2004) therefore critically depends on CSPs embracing the systemic goals for which they are designed (Bryson et al. 2016), on the validity of the proposed intervention (Babiak 2009; Liket et al. 2014) and on the appropriate monitoring and evaluation techniques to keep track of evolving insights of progress (Patton 2011; Patton et al. 2016).

Many CSPs have started to use Theories of Change (ToCs) as an explication of the assumptions underlying the intervention strategy. The ToC approach potentially differs from other planning tools of social programmes such as the Logical Framework and Result Chains in that ToC reflects a Programme Theory—the rationale *why* outcomes can be expected and what might undermine causal relations (Clark and Anderson 2004).^2^ The ToC concept surfaced in the 1990s in the rapidly developing field of impact evaluation for sustainable development (Jackson 2013; Weiss 1995). It came from a dissatisfaction with the evaluation practices of the time, the limited understanding of complexity and a call for better informed project planning (Vogel 2012). The ToC approach was argued to minimise the ‘attribution problem’ in two complementary ways: by drawing stakeholders into a ‘dialogue about how and why proposed actions will generate desired outcomes’, ToCs would result in ‘a greater confidence in attributing subsequent changes to previous specified actions’ (Sullivan and Stewart 2006, 180). Secondly, by involving more stakeholders, contextual elements can be more easily included in the ToC (Blamey and Mackenzie 2007), which enables evaluators to better attribute successes to the intervention and draw learnings when no impact is achieved (Weiss 1995). The ToC is as such an improved version of the logic framework and does not only illustrate the sequence of inputs and outputs/outcomes, but also formulates and argues the underlying hypothesised links between inputs, outputs and contextual dependencies which helps to develop a rigorous monitoring and learning plan for improvement (James 2011).

Proper ToCs ‘reduce complexities by creating complexities’ (Schneider et al. 2017). But can collaborative complexities be sufficiently captured before the start of a systemic change-oriented partnership project? Complexity and systems theory literature stress the importance of multi-stakeholder decision-making processes (e.g. Maani 2017) to do justice to the complexity of the issue, but without specific reference to the sectoral origins of the problem. Systems thinking is more about factors and forces than about actors and sectors. The evaluation field that applies complexity constructs takes a comparable perspective—without reference to the issue whether the partnering configuration matches the societal problem. The dedicated CSP literature acknowledges that more complex (systemic) problems require more multi-stakeholder approaches (Austin and Seitanidi 2012) and probably more complex partnering configurations. What this actually entails in terms of problem diagnostics and the choice for partnership configuration remains largely unspecified. Designing ToCs for complex CSP projects faces at least two challenges. Firstly, the process of formulating a ToC can be ‘unsettling or even threatening’ (Weiss 1995) and can only succeed if practitioners adopt an open learning approach (Vogel 2012) and critically reflect on assumptions and interests. Secondly, participants face the challenge of understanding the approach itself. The ToC literature lacks sufficient consensus and common terminology, creating a major obstacle for its successful adoption. The lack of unity in the ToC literature can be explained by the large variety of purposes (Michie and Prestwich 2010; Stein and Valters 2012) and the wide scope of anticipated benefits, which sometimes results in conflicting requirements on how a ToC should be constructed and maintained (cf. Mason and Barnes 2007). The formulation of a ToC as an intervention strategy is often superficial as practitioners fail to make it an integral part of the process intervention development (Michie and Prestwich 2010). Moreover, it is often difficult to assess the impact of the degree of complexity on the outcome of the partnership and to involve all parties in the same way. Where the emergence of unintended outcomes is inevitable, responsiveness is anticipated to be key to success for complex interventions (Davies 2004, 2005), but how these effects can be mapped in the process of partnering has barely been addressed. Therefore, the purpose of this paper is to offer a framework and process by which partnerships can construct ToCs that are sensitive and adaptive to the level of complexity that they are facing, thereby introducing the notion of Complexity-Sensitive ToCs.

One way of approaching this comes from the (related) evaluation field. Increasingly, developmental evaluation techniques have been introduced such as adaptive cycles to support innovation initiatives (Patton et al. 2016; Westley et al. 2006) that are aimed at addressing complex and/or wicked problems. However, most of these insights and techniques have not yet been applied to specific CSP projects. Patton (2011), for instance, acknowledges that complex social innovation processes can be framed around different intensities of collaboration that can change over time. But these relatively unspecified multiple-stakeholder engagement processes are approached in the literature with an emphasis on internal decision-making challenges to enhance the performance of the partnership (cf. Maani 2017) rather than on its impact on systemic problems. The basic theory of action is characterised by Patton (2011: 245) as ‘bring good people together and good things will happen’. This seems particularly distant from the reality of actual CSP practice. The most elaborate developmental evaluation inquiry framework pioneered in a concrete partnership project (the Global Partnership for the Prevention of Armed Conflict) has become that of ‘outcome harvesting’ (Wilson-Grau et al. 2016). This approach still belongs to the performance-based school of evaluation, which is different from the impact-oriented approach needed to address complexity and systemic change over time, with due reference to the impact of CSPs beyond output or outcome (Van Tulder et al. 2015).

Practitioners in the CSP area aimed at systemic change are struggling with how to approach the proper use of ToCs—particularly at the start of a project. An evaluation by Aidenvironment of over fifty cross-sector development partnerships initiated by the Dutch Ministry of Foreign Affairs suggested that the ‘sophistication’ of the adopted ToC provided a key predictor for the success of the intervention (Kessler et al. 2010). However, what ‘sophistication’ entailed remained unclear. Nevertheless, follow-up initiatives on partnerships in food and water security by the Dutch government made a sophisticated ToC (based on a logic framework) an important precondition for the approval of the project proposal. A survey conducted among the participants of these initiatives confirmed that partnerships experienced considerable difficulty in specifying a ToC in their proposals: 50% considered it helpful to specify a ToC, 33% disagreed, 55% found it difficult to specify a ToC, and 32% did not. When asked about their experience with formulating a more straightforward technique like the ‘results chain’, respondents were less ambiguous: 70% considered it helpful, while only 34% found it difficult (PrC 2014). The Dutch experience shows that a major challenge for publicly funded CSPs has been the relatively limited tolerance for ambiguity of partnership strategies on the side of the funders.

Funders often expect linear action plans and hold CSPs accountable for the realisation of intended outcomes rather than for fast learning and quick adaptation to new insights, which developmental evaluation techniques would propose as a locus of accountability (Patton 2011). In response to criticism on the attributable value of CSPs, performance-based contracts have increasingly been used for partnering projects. When CSPs are held accountable to a ToC that was developed in an explorative brainstorm session, it is likely that both funder and practitioner will be disappointed with the collaboration. The realisation of this problem has made donor organisations such as the UK Department for International Development (DFID) as well as the International Monetary Fund increasingly recognise the need to challenge dominant methodologies in the development sector, to pay more attention to the complexity of systems and to consequently design more adaptive approaches to change in which ToCs are not used as ‘planning’ but as ‘navigation’ tools (Ramalingam et al. 2014). This line of thinking has been advanced in the strategic management literature at the individual level of organisations when faced with systemic and responsive processes (Stacey and Mowles 2015). But most of this line of thinking has not been applied to the specific problems of transformational CSPs aimed at systemic change.

CSPs face conceptual and analytical challenges when designing and applying ToCs for systemic change because systemic change processes do not have clear solutions. At the same time, it is widely acknowledged that effective partnering serves as a critical requirement for transformational change. The risk is that CSPs might not live up to their transformational potential in addressing systemic change because of their inability and/or unwillingness to consider the complexity of the societal problems they aim to address. Consequently, many CSPs fall short of designing a sufficiently sophisticated intervention strategy, construct partnership configurations that do not include key actors to address the issue, or fail to define partnership goals that can be monitored and governed appropriately. A poorly defined and applied ToC in CSPs aimed at systemic change might even become part of the problem rather than part of the (intended) solution. Complex partnering initiatives require a more Complexity-Sensitive approach to working with ToCs. Such a novel approach should support partnerships in dealing with three key partnering challenges, which thus constitute the three key requirements for a Complexity-Sensitive ToC. First, a Complexity-Sensitive ToC needs to support partnerships in adequately appreciating the level of complexity under which a partnership operates, which in fact derives from the systemic problems that partnerships address. Second, this appreciation of complexity should inform (fine-tune) the partnership configuration. Third, a Complexity-Sensitive ToC should support partnerships in aligning an appropriate learning strategy, which should be more reflective and adaptive as partnerships face higher levels of complexity.

The remainder of this paper is organised as follows: Section “Matching Theories of Change with the Transformative Capacity of Partnerships” identifies the components of a Complexity-Sensitive ToC. We develop a complexity alignment framework that informs practitioners on how to apply complexity thinking for each of the three partnering challenges. Section “Issue Complexity: The Need for a Situation Recognition Heuristic” deconstructs and maps the complexity of the target issue of the CSP. We define eight components for a more dynamic ToC for CSPs, which can be seen as both a process and an outcome: it is a process of critical reflection on the hypothesised causal relations and underlying assumptions of an intervention strategy (program theory) that explains why an intervention can be expected to generate the intended change. Section “Partnership Configuration: Aligning Selection and Ambition” illustrates the kind of learning and evaluation challenges specific partnership configurations face. Section “Implications for Partnering Practice” then describes how these components play out differently during consecutive phases of the partnering cycle: initiation, planning and design, realisation and sustaining. We formulate propositions that can frame further research on the two related dimensions of a Complexity-Sensitive ToC: issue/design propositions that relate to the design aspect of the partnership and process/learning propositions that relate to the type of learning strategy that the TOC should support. Section “Conclusion and Further Research” concludes and defines areas for further research.

## Matching Theories of Change with the Transformative Capacity of Partnerships

The aim of this section is to discuss the purpose, the key components, and timing challenges of a Complexity-Sensitive ToC for CSPs aimed at systemic change. We build on the literature in three streams of research: cross-sector partnering, theory of change and evaluation. In the CSP literature, it has been widely suggested that the initiation phase of a partnership is critical for consensus building on problem definition, goals and intervention (Glasbergen et al. 2007; Innes and Booher 1999). The consideration and inclusion of both internal and external stakeholder perspectives is considered an important determinant of the quality of formal and informal agreements made during formation of a partnership (Bryson et al. 2006). Initial agreement on the collaborative strategic plan may affect the extent to which partnerships can exploit their collaborative advantage (Huxham 1996). The CSP literature suggests that partners can only fully exploit the synergies of working together if they agree on a common vision, mission and objectives (Clarke and Fuller 2010). However, the more complex the challenge is, the more difficult this ambition is during the first phases of the partnership. Differing institutional logics between partners can complicate goal alignment (Erakovich and Anderson 2013). Objectives, ideas, interests and values will probably differ or may even conflict (Seitanidi et al. 2011). If partners fail to agree on a shared purpose and a common analysis of the problem, it is very difficult to agree to a plan of action (Huxham and Vangen 2004; Austin and Seitanidi 2012) and on a mission, vision and goals (Westley and Vredenburg 1997). Awareness that the organisation needs others to solve the problem (Bryson et al. 2006) provides another condition that affects the form and content of a collaboration agreement (and ultimately its impact). Glasbergen’s (2011) ladder of partnership outcomes relates to the degrees of complexity. Overcoming differences and building trust is the first level of partnership outcomes.

In the CSP literature, the scope of societal change that CSPs can achieve has been referred to as the transformative capacity of the partnership (Austin and Seitanidi 2012; Seitanidi et al. 2011). Transformative capacity is determined by the motivation of the partners, the issue addressed and the level of benefit a partnership can achieve for its partners (Selsky and Parker 2010). But transformative capacity is also dependent on the dynamics of the partnering formation process and the chosen partnering configuration (Clarke and Fuller 2010; Innes and Booher 1999). Particularly, the degree to which partners can *build* consensus on goals, shorter and longer-term effects (first-, second-, and third-order effects of the intervention) affects transformative capacity. The process of agreeing on a collective ToC is expected to build trust, legitimacy and manage conflict within a partnership. These elements are proposed to be key ingredients of a successful CSP (Bryson et al. 2006). Outcomes depend on emergent implementation as partnerships go through consecutive stages (Glasbergen 2011) in which they move over time from inputs and outputs at the actor level toward outputs at the structure and systemic level.

ToCs facilitate alignment on problem definition and intervention objective during the formation phase (Innes and Booher 1999; Retolaza 2011) and can therefore be an appropriate tool to enhance the transformative capacity of CSPs. ToCs can enhance the impact of interventions (Vogel 2012). They stimulate practitioners to include various stakeholder perspectives when theorising how a complex intervention can best achieve its objectives (Innes and Booher 1999; Weiss 1995). A Complexity-Sensitive ToC approach should thus facilitate partnerships to learn how internal processes, systemics and capabilities can be best developed and adapted to the complexity level of the change process that a partnership aims to achieve (Aragón et al. 2010). Rather than monitoring whether the intended change is observed, a highly complex change process requires evaluators to trace the configuration of critical causal pathways and observe how these develop over time. Such an adaptive approach to ToCs tends to limit the attribution dilemma (Sullivan and Stewart 2006) and enhance ‘learning by doing’, independently from whether the intervention turns out to be a success or a failure (Vogel 2012). Finally, Complexity-Sensitive ToCs facilitate the aggregation of evaluation results into a broader base of programme knowledge (Weiss 1995). Putting ToCs to the test of practical application has been called causal process tracing (Vellema et al. 2013). It allows researchers to refine programme theory through a comparative case analysis of partnerships that uses the same type of intervention to reach a common objective.

A Complexity-Sensitive ToC supports systemic change by shifting strategy from a planning approach characterised by single-loop thinking towards a theorising and learning approach characterised by double- or triple-loop questions of effectiveness (Jackson 2013; Stacey and Mowles 2015). The appropriate use of a ToC should therefore be contingent on the partnership configuration for which the ToC is developed and for the kind of learning and reflection it aims at, which is ultimately related to the degree of complexity for which it is designed. A Complexity-Sensitive ToC is holistic as well as dynamic: when used for highly complex systemic ambitions, it leaves room for an evolved understanding of how change happens.

The delineated approach to Complexity-Sensitive ToCs for CSPs is further reiterated by the developmental evaluation practice which was introduced to better facilitate more complex evaluations settings characterised by uncertainty and emergence (Patton et al. 2016; Westley et al. 2006). This evaluation approach differs from summative (aiming to assess the overall merit of a programme) and formative evaluation (aiming to support the improvement of a programme) approaches by being utility focused. Developmental evaluation aims to help social innovators explore possibilities for addressing major problems and needs (Patton 2011). In this novel approach, the interests of the implementer and evaluator are aligned as they both seek to optimise impact where the developmental evaluator helps to guide adaption of the strategy to dynamic realities in complex environments. The accountability focus thereby shifts from proving the effectiveness of a model to the ability of the innovator (or partnership) to be true to its vision and find strategies that realise the intended change (ibid). Developmental evaluation guides action in complex environments. The developmental evaluation approach posits that as the environment of an innovation or intervention becomes more complex, it becomes more important to learn by doing and to collect informative data which can guide action. When confronted with high degrees of complexity, the ToC should be seen both as an outcome and as a process: ToCs are both *theories* of how change can be realised and *approaches* to making these theories better informed through testing them in practice.

Understanding ToCs as a process implies that they can become ‘smarter’ or better informed over time, and particularly during the realisation phase of partnerships. The time factor is a much-overlooked aspect in the ToC literature. While guidance is provided on how to formulate a ToC (Retolaza 2011; Stein and Valters 2012; Vogel 2012) and on how to graphically represent highly complex ToCs (Davies 2004, 2005; Rogers 2008), the literature (academic and non-academic) falls short of providing any actionable instructions on how to evolve or refine ToCs during implementation (Retolaza 2011). Every ToC displays a change process with growing uncertainty and complexity as change unfolds. Tracking whether a partnership delivers on its planned activities (e.g. training farmers) constitutes a straightforward, single-loop learning exercise. Establishing whether these activities do indeed translate into the anticipated outputs (increased knowledge) and outcomes (adoption of practices) is a more complicated, double-loop learning challenge. However, establishing whether the partnership contributes to the intended change (increasing productivity, income and resilience) is generally understood as a very challenging, yet much needed, triple-loop learning (Van Tulder et al. 2015; Wadell 2011). While acknowledging that some discussion on the exact nature of triple loop learning processes is on-going (Keen et al. 2005; Tosey et al. 2012), we apply insights from Armitage et al. (2008) to partnering in the following manner: single-loop learning relates to the revisiting of partnership (learning) strategy, double-loop learning entails a revisiting of the situation, whereas triple-loop learning involves reconsidering the configuration of the partnership. Both the time needed for outcomes and impact to unfold, and the challenges faced in attributing these changes to a partnership make it very difficult for CSPs to obtain timely information on whether they are on the right track to have an impact. Particularly when aiming for highly complex systemic change, it is thus of vital importance for partnerships to identify early signals of whether the partnership is on track towards the intended change. This can be done by monitoring the most critical assumptions (or hypotheses) of the ToC and starts with defining the nature of the problem.

**Fig. 1:**
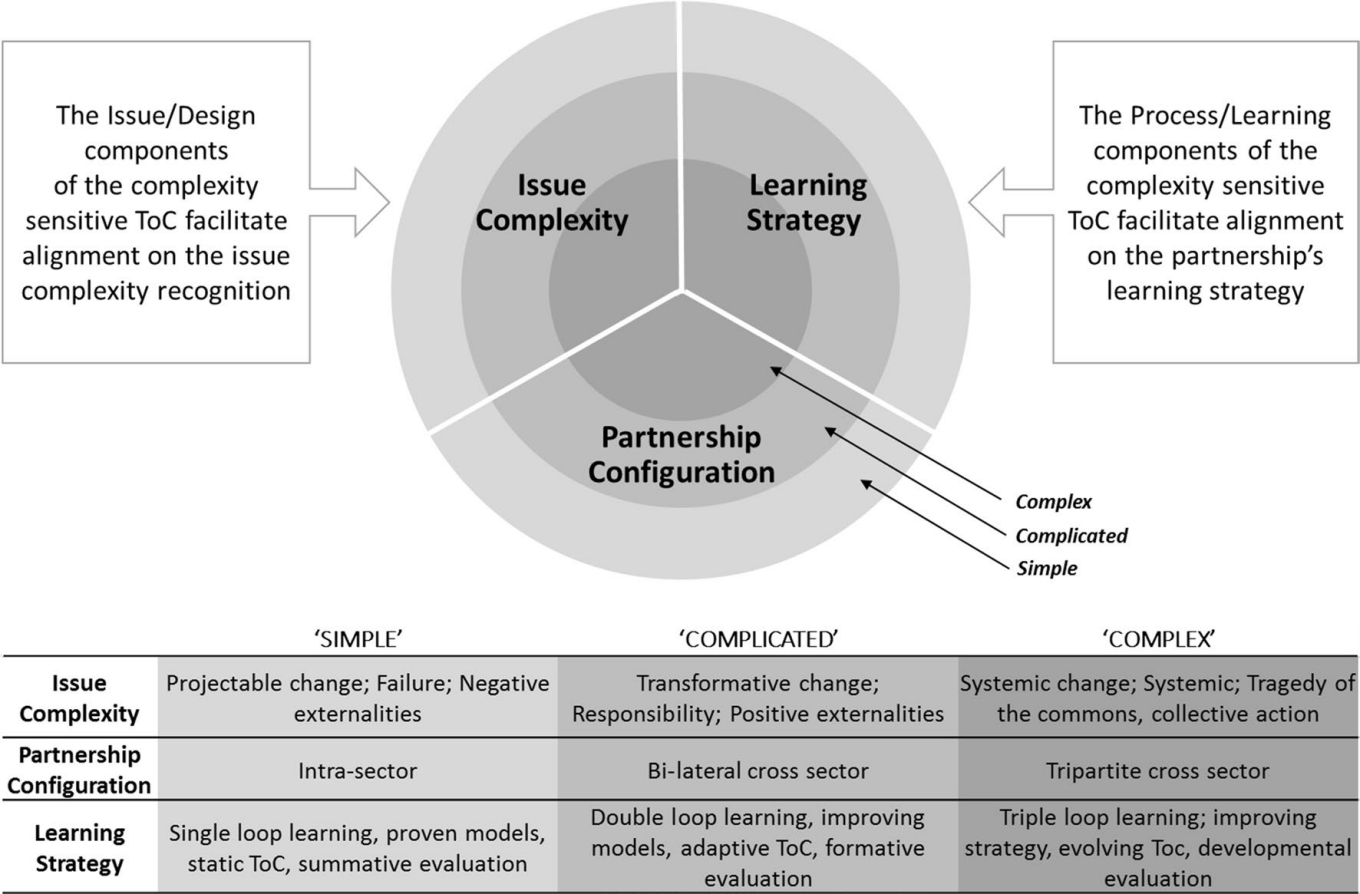
Complexity alignment framework for CSPs

In conclusion, addressing the three key requirements of a Complexity-Sensitive ToC implies a framework in which the various degrees of complexity can be aligned with the various uses of ToCs in terms of accountability, learning (loops), leading questions, change process and evaluation approach. Figure 1 shows the resulting complexity alignment framework. It has three levels of complexity—slightly tweaked from the complexity and wicked problems literature—as basis for alignment. The wicked problems literature distinguishes between ‘simple’, ‘complex’ and ‘wicked’ problems (Jordan et al. 2014; Rittel and Webber 1973). The complexity literature distinguishes between ‘simple’, ‘complicated’ and ‘complex’ (Davies 2004, 2005; Rogers 2008). Rogers’ operationalisation of ‘complex’ is comparable to the category of ‘wicked’ in the former approach. Patton (2011) also refers to ‘chaos’ when moving beyond complexity. Integrating these two streams of literature implies that the classification of ‘complex’ is used with an acknowledgement that even within this highest level of complexity a more fine-grained distinction can be made between problems that are complex, highly complex or wicked, and extremely complex or chaos. In this paper, we bulk these levels of complexity together because they all require a highly adaptive approach when working with ToCs.

With the growing complexity of an issue, the ToC approach changes in ambition and elaboration (Fig. 1). Figure 1 depicts the alignment framework and shows the link between the level of complexity of the issue, the partnership configuration and the partnership learning strategy. The more complex the intervention of a CSP is, the more diverse and thus more challenging a partnership will be, and the more adaptive the ToC should be. This implies that partnerships face more challenges as they address more complex issues, and that it will be more difficult to understand the issue, more difficult to collaborate with a more diverse group of partners, and more difficult to be flexible and honest when engaging in an adaptive, triple-loop learning strategy.

The ToC approach can support partnerships achieve an alignment of the ambitions, the partnership configuration and the intervention and learning strategy. Based on comparable holistic approaches to ToCs (Retolaza 2011; Stein and Valters 2012; Vogel 2012) and applied to insights into success factors of CSPs emphasising iterative learning processes, we can now identify eight key components of a Complexity-Sensitive ToC for CSPs. Half of these relate to design questions, i.e. whether the partnership adequately appreciates the complexity of the target issue and can align the partnership configuration appropriately. The other half relate to the process and learning components, i.e. whether the TOC allows for appropriate monitoring and evaluation during the partnering process.

Issue/Design Components:

1. *A problem and context* analysis investigating underlying causes of the problem, its context, and the degree to which the problem can be perceived as systemic.
2. *A stakeholder analysis* identifying key stakeholders both in terms of being part of the solution (coalition of the willing) or being part of the problem (coalition of the needed).
3. *An analysis of the intended change,* ensuring goal alignment and a collective appreciation of the level of complexity of the intended change.
4. Critical reflection on *assumptions* underlying the ToC, determining whether these assumptions are grounded in evidence, practices or whether they need to be validated during implementation.

Process/Learning Components:

5. *Intervention strategy and markers for change,* using a backwards mapping approach to identify the best strategy and set-up of relevant monitoring structures.
6. Reflection on the *critical conditions,* determining the risks and potential changes in the enabling environment that cannot be influenced, but could affect the ToC.
7. A *reflective approach,* aligning expectations that the ToC is likely to evolve. Will the partnership ask evaluators to prove the validity of the ToC based on the results of the partnership, or will the partnership adjust and drastically change its ToC as the partnership unfolds?
8. A *graphical presentation*, depicting an easily understood representation of the ToC. Visual strategy mapping (Bryson et al. 2016) aids in action research and mutual learning projects. Narratives, combined with strategic mapping techniques, create frames.

The following section introduces a situational heuristic to support partnerships in appropriately appreciating the level of complexity of the target issue. This can support CSPs in developing the four issue/design components of a Complexity-Sensitive ToC. Building on this heuristic, we explain how the partnership configuration should be aligned with the ambitions of the partnership. The consecutive section provides guidance on how CSPs can develop and work with Complexity-Sensitive ToCs though four stages of partnering.

## Issue Complexity: the Need for a Situation Recognition Heuristic

**Fig. 2:**
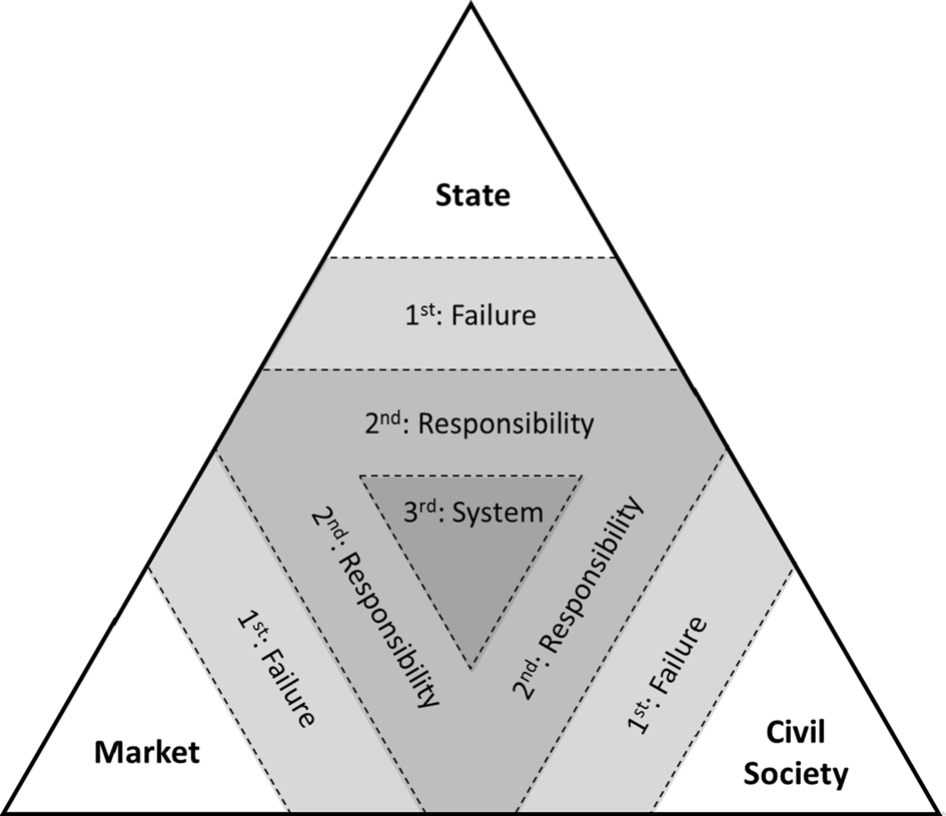
Societal layers of complexity

The four issue/design components of a Complexity-Sensitive ToC support partnerships in dealing with the first alignment challenge: how to come to a better understanding of the nature and sectoral origins of the target issue, thereby appropriately appreciating its level of complexity. We propose to develop what Patton (2011) calls a ‘situation recognition heuristic’, which for the specific purpose of transformative CSPs can be based on collective action and public goods approaches. CSPs are not cross-sector by chance—an important diagnostic tool relates to the relationship of the partnership with each of the societal sectors. We can call this societal triangulation. Section “Matching Theories of Change with the Transformative Capacity of Partnerships” discussed the sectoral origins of cross-sector partnerships linked to the type of change needed: first-order (intra-sectoral), second-order (bi-sectoral) or third-order (systemic) change. The most appropriate partnership configuration should be aligned with the type of problem it addresses (Vurro et al. 2010). Most of the partnering studies distinguish three sectors that define the sources of the partnership: states (governments), communities (the plural sector) and markets (corporations) (Seitanidi and Crane 2014,3). These three sectors of society each deliver/supply/produce complementary goods and services and thereby add complementary value to society. Public good theory explains the differences between each of these societal spheres: markets produce private value, civil society generates social value, governments organise the provision of public value (Samuelson 1954). The need for systemic change increases the more these sectors collectively fail or leave considerable gaps in addressing any specific issue. For each of the societal sectors, systemic change involves different partnering approaches across sector lines: in the private sector, NGO-firm partnerships (e.g. Austin and Seitanidi 2012), and in the public sector, new forms of collaborative governance (Bryson et al. 2015). Ideally, the sectors complement each other, making sustainable development into a balanced development (Mintzberg 2015). If any of these sectors do not function properly, institutional voids appear. The bigger the void, the greater the complexity/wickedness of the problem and thus the greater the need for systemic change. Specific sources of societal/systemic complexity can therefore be threefold (see also Section 1): (1) institutional failure from within a sector (Kolk et al. 2008), (2) lacking responsibilities that transcend the scope of influence of individual societal sectors (Clarke and Fuller 2010; Selsky and Parker 2005) and (3) insufficient collective action taken by all three sectors at the same time (Bryson et al. 2015; Van Tulder and Van der Zwart 2006) that might hamper the innovative and transformational capacity of the partnership (Patton 2011) or the creation of positive systemic effects (Googins and Rochlin 2000). Three orders of change define the degree of complexity addressed by any type of partnership (Fig. 2).

### First-Order Change Challenges

The first layer of societal issues is relatively simple. It is the failure of each sector to efficiently deliver its principal value to society. This is a common and universal phenomenon that is nevertheless often poorly recognised as a source of sustainability problems (cf. Nooteboom 2014). In ethical and legal theory, this dimension is also referred to as the ‘fiduciary duty’ of an organisation in a narrow sense—the core function of organisations towards their primary stakeholders/principals (customers, owners, members and employees). Firms, for instance, do not supply sufficient (private) goods or services to the existing market, although they should be perfectly capable of doing this (e.g. pharmaceutical firms that do not supply medicine to sick people that lack the purchasing power; food companies that contribute willingly to obesity; gambling industries that target vulnerable consumer groups). They create (and suffer from) market failure. Civic organisations that do not adequately organise the mutual support of citizens provide a poorly managed social (or ‘club’) good. Corrupt civic leaders can create civic failure (Kolk et al. 2008). The greatest source of human rights violations occurs within communities themselves. The ability of the state to develop proper laws and provide sufficient public goods can be perverted by kleptocratic rulers who use tax income to fill their own bank accounts. Governance failure occurs if governments are not able or willing to formulate and/or enforce laws. Failure creates negative externalities for other sectors of society. The analytical complexity of these problems is primarily related to the inability or unwillingness of the people involved to coordinate their activities with others in the same sectors and restore trust in the public perception of this sector.

### Second-Order Change Challenges

The second layer of societal issues is more complicated. It relates to the unwillingness of a sector to extend its influence beyond its primary stakeholders, its core activities, and take up a more broadly defined fiduciary duty (that extends towards secondary stakeholders as well). Rather than dealing with ‘negative externalities’, this dimension deals with the stimulation and utilisation of ‘positive externalities’. Firms can extend their positive influence on society by targeting latent demands and needs of society (e.g. products for poor people for which no market yet exists). They can do this through philanthropic (CSR) activities, but more is needed if they want to link this to their core capabilities. Civic organisations can accept responsibilities beyond their own community or club. These include ‘social enterprises’, international solidarity through mutual support activities or an engagement in advocacy action in which civil society organisations target other parties to take up their responsibilities. For states, this means facilitating or endorsing activities through subsidies or other indirect measures by which they influence society other than through laws (mandating). The accumulation of insufficient actions of sectors to take up responsibilities increases the degree of complexity. The danger of ‘crowding out’ looms large in activities where organisations accept too much responsibility, which can result in unintended (negative) effects on the activities of other actors, which in turn increases the complexity of the problem. Global Goals that are primarily related to the well functioning of markets or value chains (for example, SDG12 on responsible consumption and production, or SDG2 on food security) potentially require only well-aligned private sectors and very limited government involvement. Bilateral partnerships between firms and NGOs can suffice, certainly if governments take up their prime role in providing a proper legal environment. Global Goals that require infrastructure (for example, SDG9 on innovation and infrastructure) deal with the interface between public goods and their (partly) private provision. Bilateral partnerships between governments and firms (classic PPPs) are the most obvious partnering configuration on these issues.

### Third-Order Change Challenges

The third layer of societal issues is the most difficult to address. They represent that part of the societal set-up that requires the participation of all actors in society. However, these actors may not feel responsible and primarily see the risk of getting involved. The institutional void is substantial, because there is no formal organisation that can coordinate or take up responsibility. This is the case for almost all global public good issues. Addressing climate change challenges requires multi-sector partnerships that can take the nexus of climate and sustainable development into account (Pinkse and Kolk 2011). It is also the case for most economic growth topics, which need common action beyond individual responsibilities to enact a minimum level of social, economic and ecological regulation. In the SDG agenda, the ambition for ‘inclusive green growth’ requires a nexus between almost all SDGs. Collective action should provide ‘common goods’ that go beyond private, public or social goods. Risk-taking requires risk-sharing in these areas. Such a level of complexity also creates the greatest risk of the ‘bystanders’ effect: all parties have to take on responsibility, but they find the risk too high to do it on their own. The institutional void that is created by this type of problem is also known as the ‘*tragedy of the commons’* which requires innovative governance and partnering arrangements (Ostrom 2010). Tragedy of the commons problems are defined as ‘super-wicked’ (Levin et al. 2012). In complexity theory, references to the challenge of ‘chaos’ can be found (Patton 2011). In leadership literature, forming the right coalitions for collective action challenges is referred to as proactive leadership. In the business literature, systemic change approaches and disruptive innovation are likewise discussed in terms of proactive (Torugsa et al. 2013) or shared value creation strategies (Porter and Kramer 2006), which in their most radical form requires the creation of new (proto) institutions.

The complexity of issues, in societal terms, can have three origins: civic (or communities), public (or state), market (or corporate). Table 1 presents a complexity diagnostic tool that assesses issue complexity.

**Table 1:**
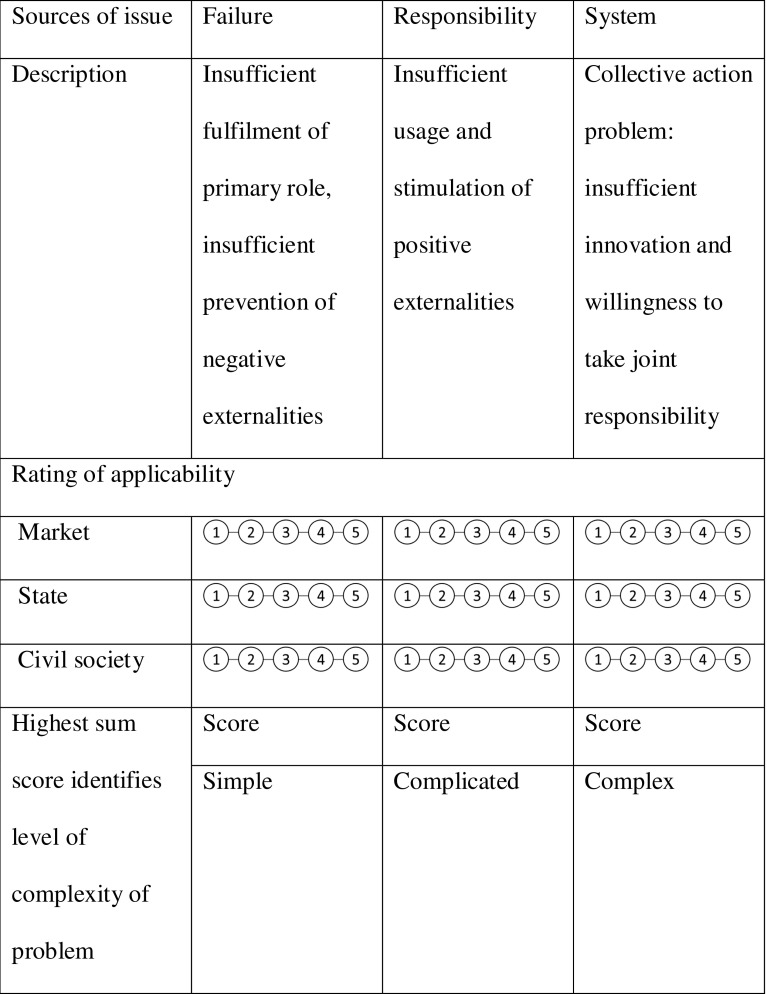
Issue-complexity diagnostic tool

From an institutional perspective, leading questions about the systemic change potential of partnerships thus depart from the question whether the partnership configuration helps one of the three basic societal sectors (1) to address some of its failures (e.g. helping to defend its legitimacy towards other sectors), (2) to help other sectors to complement their activities (e.g. sharing responsibilities and expanding legitimacy) or (3) to fill in the natural voids that are left by all sectors and can only be addressed by sharing and taking risks and extending responsibilities (Table 1). A dominant partnership configuration can be found for each type of problem (Van Tulder and Pfisterer 2014). Partnerships aiming to tackle the negative externalities and reputation effects originating from the inadequate action of individual actors in a sector are best tackled by intra-sectoral partnerships. More transactional partnerships can also aim at restoring trust, which is the logical consequence of overt failure. Responsibility-oriented partnerships are more complex, but the positive externalities that can result from actors taking up responsibilities beyond their core activities can best be reaped and facilitated through bilateral partnerships. Partnerships that are aimed at collective action, address super-wicked problems or deal with the widest institutional voids are likely to require tripartite partnerships (Pinkse and Kolk 2011). Likewise, the goals of the partnership will broaden, depending on the nature of the problem. Bryson et al. (2016) distinguished between different ‘goals systems’: with a bearing on the whole of society. They identified ‘core goals’ (either shared or embraced by an individual organisation related to its core activities); ‘beyond core goals’ (including positive and negative public value goals related to positive and negative externalities); ‘negative avoidance goals’ (avoiding reputation effects and disasters related to direct risks); and ‘not-my-goals’ (goals for which nobody wants to be held accountable for their achievement, even though they can be supported). This runs parallel to the societal degree of complexity that partnerships are supposed to address. A clear assessment of the complexity of an issue can be made if the scores converge around one column. For instance: high on failure (15), but low on responsibility and system/collective action problems (3–6), then relatively focused alliances within sectors present a logical approach. If one sector creates complexity by scoring high on simple, complicated as well as complex challenges, the issue complexity is primarily related to making that sector more active. If the scores widely diverge (both along rows and columns), the complexity—even the ‘wickedness’—of the issue increases, as well as the need to apply third-order learning loops. Complexity does not only increase along the sources of the issue (the vertical columns), but also along the degree of alignment that can be achieved among the three societal sectors (the horizontal rows). Depending on the individual scores, the intensity of participation in partnerships can differ among participants.

## Partnership Configuration: Aligning Selection and Ambition

Table 2 provides examples of the possible functions of the three types of partnerships and their learning and evaluation challenges. In the case of the market sector, firms can measure the extent to which they contribute (1) to the provision of private goods (prime area of the market sector), (2) to the provision of social goods (prime area for civil society) and (3) public goods (prime area of the state). In case of insufficient provisions of private goods, the partnership can help to compensate for its failures. This is the case, for instance, with firms that do not reach the ‘bottom of the pyramid’ (BoP) with their products for a variety of reasons. Product development partnerships are a way to deal with the failures that companies face in attempting to serve the BoP by creating scale and/or innovation. The wickedness of the failure problem is largely intra-sectoral, and the partnership challenge largely depends on the ‘fit’ between partners from the same sector (Kim et al. 2012). Intra-sectoral partnerships might therefore still be relevant. They aim at a coalition to enhance the efficiency of the sector in delivering core activities (regulatory coordination and product development partnerships).

**Table 2:** Partnership configuration and learning strategy alignment

Issue complexity	Simple	Complicated	Complex
Order of change required	First-order change	Second-order change	Third-order/systems change
Partnership objective	Filling the failure void	Extending responsibility	Extending risk-taking Collective action for systems change
Dominant configuration of partnership	Intra-sector Negative externalities	Bipartite cross-sector Positive externalities	Tripartite cross-sector Risk-taking; tragedy of the commons
Dominant goal type category	Transaction; negative public value; negative avoidance goals	Integrative; (shared) core goals; positive public value	Transformational; beyond core sector goals; not-my goals; system change
Main initiator of partnership			
Markets	BOP (access to) medicine, product development partnerships (PdPs)	Roundtable on sustainable palm oil/soy; marine stewardship council; food and nutrition security	Climate coalitions; fair income distribution coalitions (OECD)
State	Donor coordination partnerships; fair taxation coalitions; NATO and other military alliances	Water operator partnerships; education partnerships; health; security partnerships	Water and sanitation; access to energy; access to justice; biodiversity partnerships
Civil society	Obesity partnerships; human rights coalitions; urban development partnerships	Advocacy partnerships; food security; gender partnerships; trade union rights	Poverty, economic growth coalitions
Learning loop	First order: correcting routine errors, improving the partnership strategy	Second order: correcting values and policy errors, adjusting partnership strategy due to better understanding of the issue	Third order: correcting design errors by rethinking the partnership configuration
Type of evaluation	Summative	Formative	Developmental

Bilateral firm-NGO partnerships, on the other hand, are often temporary and/or aimed at trust-building or trust-restoring. A partnership between a company and an NGO can help the company to explore and develop innovative products for these markets. A partnership with a government can help to create the institutional conditions for developing this market (either by creating a level playing field, endorsing initiatives or subsidising innovative market-based solutions to specific societal problems such as food and nutrition security or access to cheap energy and finance). In more risky areas, in which the danger of crowding out looms large and negative externalities or ‘public bads’ are prevalent, firms have an interest in supporting tripartite partnerships in which all societal spheres are equally represented with the aim to align goals and create action in areas where this is lacking due to a wait-and-see attitude of key stakeholders. Stadtler (2016) developed an integrative evaluation framework for this purpose.

A considerable number of existing CSPs are, however, so-called coalitions of the willing. They are primarily aimed at solving failure or lacking responsibilities in an unbalanced society. At the same time, they are presented as ‘system change’ partnerships. This can explain why they do not deliver (Pattberg and Widerberg 2016), not only because they crowd out other more relevant ‘coalitions of the needed’, but also because they have probably not made the right complexity analysis and were too optimistic (or perhaps too pretentious) about their ability to design a proper intervention strategy and ToC. Profit–non-profit partnerships that aim at the provision of public goods may crowd out governments, with inadequate governance as a result. Public–private partnerships that aim at the provision of community goods can remove important incentives of communities and citizens to take up responsibility for their own interests. Government–NGO partnerships, aimed at the provision of private goods (often subsidised), disrupt the functioning of markets and can thereby limit efficiency. Systemic change is most needed when all three sectors at the same time fail in taking up their fiduciary duty, while not taking any additional responsibility or risk. A skewed distribution of failure reiterates the necessity for rebalancing society and radical renewal (Mintzberg 2015) in which the initiative of the change often lies with that sector that functions relatively well.

## Implications for Partnering Practice

Complexity alignment implies that levels of complexity of the problem (or intended change), the partnership configuration, and the partnership and learning strategy are aligned. The Complexity-Sensitive ToC approach supports partnerships in aligning these components and in evolving their understanding of the issue, their strategy and perhaps even evolving the partnership configuration throughout the partnering process. As partnerships aim for the higher hanging fruit of systemic change, the more likely it is that they will have to revise their ToC and update it several times. When partnerships address a complex problem such as poverty while working with a static TOC, which focuses on the operational learning challenge and summative evaluation approaches, they are likely to be criticised for the impact that they can attribute to their efforts. However, also tripartite partnerships with a much larger sensitivity for complexity, that aim at updating their ToCs as their understanding of the issue progresses, will face scrutiny if their funders keep them accountable to the ToCs they had formulated during the initiation phase of the partnership (Section “Introduction: Designing Collaborative Interventions for Systemic Change”). Hence, developing the eight components of Complexity-Sensitive ToCs is only a first step and does not automatically make ToCs fit for purpose. These eight components are likely to demonstrate evolving functionality during four more generic phases of the partnering cycle: initiation, planning and design, realisation and sustaining (Tennyson 2003). This section formulates propositions on how the four issue/design and four process/learning components of a Complexity-Sensitive ToCs can support the partnership during each phase to enhance the transformative potential of CSPs (as discussed in Section “Matching Theories of Change with the Transformative Capacity of Partnerships”).

### Phase 1: Initiation

During the initiation phase, the discussion on the ToC focuses on aligning a common aspired impact and including key stakeholders in the partnership configuration. In most cases, the partnership will not have developed and completed all components of a Complexity-Sensitive ToC until the end of the planning and design phase

*The problem and context analysis* is most crucial during the initiation phase. The analysis should include the scope (first order, second order, third order) and trends of the problem (worsening, improving or remaining constant). Key underlying causes need to be identified. The societal triangulation tool assists in defining the nature of the problem. Partners need to be willing to share their dilemmas and failures—in addressing the problem on their own (Kolk et al. 2008). The problem statement should clarify how partners relate to the issue. Filling out Table 1 should reveal the societal sources of complexity. When aiming to address more complex issues, potential partners will voice much more uncertainty and perhaps disagreement on the problem analysis.

Equally important is the *stakeholder analysis*. In more complex environments, stakeholder mapping will help identify opportunities for collaboration (win–win) or identify conflicting interests or prisoner’s dilemmas that need to be overcome to unlock positive change. Many partnerships bring together parties that want to collaborate, but not necessarily include those parties that are directly responsible or those that could play a major role in addressing the issue. A distinction needs to be made between ‘coalitions of the willing’ and ‘coalitions of the needed’. By using the issue-complexity diagnostic tool presented in Table 1, it becomes clear whether the origins of the problem converge or diverge for each sector, which implies not only different role attribution but also increases the danger of crowding out by ill-aligned partnerships. Most existing partnerships aimed at systemic change fall particularly short of organising coalitions of the needed. This is easy to understand, considering the possible accumulation of complex problems: failure, lacking responsibility and insufficient collective action all appearing at the same time. A stakeholder gap analysis can be helpful in this case. The ToC needs to take the gap between ‘needed’ and ‘willing’ into account. Successful partnerships in conflict regions, for instance, did not originally include the (corrupt) government in the formation process, but identified it as an important stakeholder to be included later on in the process (cf. Pfisterer 2013).

After an *analysis of the intended change*, the partners can then start to align on the intended change (or the aspired ultimate impact). In simpler settings, it may be possible to identify a pathway of change that can be organised and tested by the partnership. In complex settings, reaching agreement on the intended change will not only be challenging (Huxham and Vangen 2004; Ugboro et al. 2001), but probably impossible given the complex nature of third-order problems. A key objective of the partnership can then become increased alignment and a common understanding of the issue.

During most complex change processes, the partnership will lack maturity to *identify and test assumptions* underlying the intervention strategy. Yet, there will most likely be some assumptions about the long-term involvement of the partners during the sustaining phase of the partnership. Other relevant assumptions that can already be investigated concern the desirability of the intended change. In complex settings, where partners differ greatly in their analysis of the problem, many of assumptions are likely to surface through the initial dialogues on the issue and the intended change. Thus, involving people and organisations that question key assumptions of the partnerships can be useful. It guards the partnership from tunnel vision.

*Issue/Design Proposition #1:* Partnerships that focus during their initiation phase on coming to a shared analysis of the origins of the problem and identifying whether they form a ‘coalition of the willing’ or ‘a coalition of the needed’ are more likely to succeed in generating systemic change than partnerships that primarily focus in this phase on creating a common vision, mission and objectives.

During the initiation stage, participants are unlikely to agree on any detailed *intervention strategy and markers for change* or to be able to reflect critically on the *critical conditions* for the ToC. Yet, it is relevant to start talking about ‘what will change look like’ and ‘how do we get there’. In this phase, alignment on the desired *reflective approach* towards the ToC is most relevant: regularly updating the ToC during all phases of the partnership can even become the purpose of a partnership to test, validate, develop or change the ToC as insights of the partnership evolve. In such an agreement, the ToC becomes an indicator of evolving strategies. It can also be useful to start developing a *graphical representation* of the intended change process. It is not necessary to finalise such a representation during this phase, but it can facilitate the dialogue on the ToC. Discussions on the initial components and causal linkages in the ToC graph can illustrate the alignment within the partnership.

*Process/learning Proposition #1:* If the innovative potential of CSPs is stifled by defining detailed monitoring and evaluation ambitions and defining detailed goals during the initiation phase of complex CSPs, the willingness of potential participants to share dilemmas and build up mutual trust will decrease.

### Phase 2: Planning and Design

The beginning of the planning and designing phase is marked by revisiting and fine-tuning the *problem analysis, the stakeholder analysis* and the *analysis of the intended change* to ensure that all partners are aligned. Failing to align on these fundamental components of the ToC is likely to result in resistance and conflict when designing a plan of action. Alignment includes agreement among partners, but also within partnering organisations. Support of senior management is thereby important not only for the partnership to take place, but also to set up the relevant monitoring and governance structures (Battisti 2009; Eisenhardt and Schoonhoven 1996) that can enhance the impact of the partnership through its feedback loops among the participating organisations in later stages (Van Tulder et al. 2015). During this stage, the participants can decide to invite stakeholders to the coalition to create a better fit between the problem and the partnership configuration (Vurro et al. 2010). By adding new stakeholders, the problem analysis can deepen, which in turn can help create more realistic or constructive change ambitions. During this stage, it becomes relevant to change the ambition of the partnership from one defined by negative frames to one defined by positive frames. In the past, where ‘negative’ frames prevailed for partnerships and stakeholder engagement processes, they were found to be of relatively limited effect on the success of the partnership (Warner 2006). A doom scenario triggers a sense of urgency, but creates defensive risk-avoidance partnerships. However, risk-taking partnerships are needed for systemic change. Positive frames, and jointly defined ambitions that go beyond the individual responsibility of actors, probably present a better frame to organise cross-sector partnerships (cf. WBSCD 2014; UN 2015).

Simultaneously, it is important to pay close attention to the *assumptions* underlying the intervention model and to the *critical conditions* that form potential risks for the success of the intervention. In more complex settings, such a dialogue can guide the partnership in identifying environmental factors that need to be closely monitored so that the partnership can adapt quickly if conditions change. In simpler settings, assumptions and critical conditions can inform the monitoring framework so that it will produce stronger proof on the attribution of a tested model. Some assumptions underlying the ToC can most likely already be validated through evidence from research or proven models in similar contexts. Critical conditions relate to changes in the environment (particularly relevant for complex settings), but also to dynamics within the partnership. Whether partners are loyal, share ownership and are willing to share experiences depends on power relations (Ellersiek 2011), and a lack of either trust or some degree of power balance will constitute a significant risk for the partnership. Adverse risks of unintended consequence should be considered at this stage as well, including the risks of crowding out key actors. Including all partners and external stakeholders when reflecting on the assumptions and risks is likely to significantly improve the robustness of a ToC as it will allow the partnership to utilise the wealth of knowledge present in all partners. Likewise, inclusion of different units within partnering organisations, such as local offices, is important for the embeddedness of the ToC in ‘the reality on the ground’. Asking feedback from external stakeholders will have a duel benefit of improving theory and obtaining legitimacy for the partnership.

*Issue/Design Proposition #2:* If CSPs use the design phase to frame their ambitions positively whilst critically reflecting on the risk of a poor partnering fit, then the partnership is more likely to grow into an aligned problem-partnering configuration.

In the second phase of partnering, the *intervention strategy* needs to be worked out in more detail. Firstly, the partners need to be aligned on the intervention strategy that will be put to the test by the partnership. Secondly, the partnership needs to identify and align on *markers for change* along the process towards impact. This will allow the partnership to establish an effective monitoring, evaluation and learning plan. The ToC approach sets itself apart by prescribing a ‘backwards mapping’ approach. For systemic change, it is more valuable to have only a few change indicators rather than a comprehensive set comprising many variables. What the partnership needs is an early warning system to know whether it is on the right track. An upgraded *graphical representation* of the ToC can be helpful to share ideas on assumptions and conditions. In some instances, partners might refrain from any visualisation at all and instead stick to some key principles (Patton 2011) or common rules (Rogers 2008) that guide their action (Davies 2005; Rogers 2008). Principles have the power to direct action in the face of complexity. Yet, at this stage most partnerships will benefit from developing some kind of single page synthesis summarising the ‘what’ and the ‘how’ of a partnership. In less complex settings, the graphical representation will support the development of the monitoring and learning plan. In complex settings, the graphical representation can be understood as a visual strategy map (Bryson et al. 2016), which may constitute the baseline strategy of the partnership.

It is of vital importance that all partners (including the funders) agree on the *reflective approach* and the role of the ToC in this. Evaluators should now be involved so that they can agree with the partners whether to support the partnership with summative, formative or developmental evaluation techniques. The selected reflective approach defines the most suited governance model that will guide the partnership throughout the realisation phase. A good governance structure supports the chosen strategy, for example, by bringing in expert knowledge or best practices and by ensuring that the partnership in grounded in reality (Brinkerhoff and Brinkerhoff 2011). This has also implications for transparency and accountability. Aligning on the most appropriate reflective approach will enable to partnership to manage accountability expectations both internally (e.g. with funders) and externally (e.g. with watchdog NGOs). If partnerships anticipate that the ToC will evolve, external transparency might jeopardise a constructive internal learning process, which requires internal transparency and trust-building among the participants (Molleda and Moreno 2008). Trust is built up over time which limits transaction costs, but has different connotations in different institutional spheres (Parker and Selsky 2004). In this stage, the participants build up a culture of open feedback and critical reflection which is required for fast, iterative learning. Since trust-building and goal alignment are commonly understood as the largest explaining factors of the transformative capacity of CSPs, they become critical building blocks for a Complexity-Sensitive ToC particularly during this stage.

*Process/Learning Proposition #2:* If the design phase of successful CSPs is primarily aimed at defining a reflective approach, combined with markers for change, it should allow for learning, feedback loops and trust-building.

### Phase 3: Realisation

The actual intervention of the CSP (and its effects) creates growing insights into the effectiveness of the chosen ToCs (including the partnering configuration). It also leads to a clearer understanding of the nature of the problem and the intended and unintended consequences of the CSP. During the realisation phase, the ToC supports the partnership primarily in organising progress monitoring and learning at different levels of impact. The responsibility of organising first-order learning and feedback loops lies with individual partners as they execute the planned activities and face operational challenges. Second- and third-order learning is a shared responsibility of the partnership and requires meetings to assess whether the partnership is still on track and/or needs to change course or add stakeholders. Organising a developmental learning capacity is to some extent self-steering in identifying key learning challenges, collecting informative data and feeding this back to the partnership (Patton 2011). Being adaptive to reality on the ground and revising the ToC is likely to generate resistance in the partnership and within partnering organisations. It can be frustrating for partners to have to adjust their plans, budgets or perhaps even contractual commitments more than once. It is important to be sensitive to these challenges for each of the participating stakeholders. Having a clearly defined problem definition, more so than a common goal, will contribute to the acceptance of change.

It is also important that all partners concur with the adaptations of the ToC, which will require an inclusive learning process. A condition for this is the degree of ‘institutionalisation’ of the partnership in the participating organisations, which in turn is strongly related to the support the partnership receives from top management (Van Huijstee et al. 2007). In this phase, intended strategies materialise in realised strategies, taking into account intervening development (Patton 2011; Stacey and Mowles 2015). For strategy accountability, it is important that phase changes in the ToC are well documented and include the underlying argumentation and monitoring data. This also goes for the monitoring of, and response to risks and unintended consequences. A ToC should identify assumptions and then critically reflect on the validity of these assumptions. It is the key process through which the ToC approach assures the validity of the intervention strategy. The more complex the partnership and the challenge is, the more assumptions need to be specified and monitored during the realisation process. One final element that has been reiterated in ToC evaluation research is the formulation of a ‘business case’ that defines the extent to which each of the partners can remain financially sustainable at the end of the project (PrC 2014). The definition of a business case for each participant is particularly challenging for tripartite partnerships, which include governments, NGOs and companies, each with their logic. For systemic issues, goals will probably not be aligned at the start of the formation phase (Huxham and Vangen 2004; Ugboro et al. 2001). Goal alignment then becomes a critical requirement for successful partnering processes and should be part of the intended change trajectory as covered by the ToC.

*Issue/Design Proposition #3*: The realisation phase of CSPs will be more successful if the partners make decisive intellectual steps to align the chosen intervention with the intended change (goal).

*Process/Learning Proposition #3*: The realisation phase of CSPs will be more successful if the reflective approach helps participants to become co-owners of the project and if they adequately institutionalise the intended change in their own organisation.

### Phase 4: Sustaining

The sustaining phase basically evolves around the question whether the partnership achieved the intended change. Yet, Complexity-Sensitive ToCs evolve over time. The decision to sustain the partnership depends on a more flexible assessment of the attributable impact of the partnership on the problem. Problem and process components are aligned and equally important. During this stage, the ‘ownership’ question (Clarke 2014) and the exit conditions become essential. It has been found that unclear exit conditions can negatively influence the partnership dynamics and limit the possible build-up of co-ownership. Co-ownership plays an important role in nurturing and strengthening local ownership of a partnering project (Guimarães et al. 2003). This paper argues that co-ownership also applies to the ToC, which needs to be a co-creation process of the most important stakeholders to be effective. The sustaining phase of a CSP aimed at systemic change can be considered effective if the partners can build up ownership (through institutionalisation) and can define the conditions of exit. Related to the alignment challenge (Table 1), this can be interpreted as the partners being successful in pooling their complementary resources and capabilities in addressing the root causes of the problem at the required level of complexity.

At this stage, the upgraded (and aligned) ToC will inform the partners on how the outcomes of the partnership can be sustained or scaled. When working on simpler change processes, the partnership might be able to deliver a best practice or proven model on how to generate change. Having such a well-documented model will inform the decision on replication or scaling of the model. CSPs aiming for systemic change will not be able to deliver replicable models. At best, they produce key insights (improved theories) into how change can be realised. These insights can be brought to a wider array of stakeholders to discuss what the best next steps could be. Next steps might take many different shapes including spin-off partnerships (which might build on trust and insights established by partnering), piloting innovations, policy amendments or just simply sharing lessons learned.

*Issue/Design Proposition #4:* The more CSPs gain insights into the conditions needed to effectively create systemic change through a specific partnership configuration, the more likely CSPs are to remain successful or even scale up their success.

*Process/Learning Proposition #4*: The effectiveness of the sustaining phase of a CSP depends on the sustained involvement of stakeholders in learning cycles related to ownership and exit conditions.

## Conclusion and Further Research

The expectations for ToCs in the existing CSP literature tend to be so restrictive that it is questionable whether any systemic change partnership can materialise under such strict conditions. This feeds into the critique of the transformative potential of cross-sector partnerships (as discussed in Section “Matching Theories of Change with the Transformative Capacity of Partnerships”). This conceptual and analytical problem is further aggravated by the general assertion that more complex (systemic) problems require more multi-stakeholder approaches. In this contribution, we argue that the transformative potential of a partnership is not primarily dependent on the degree to which partners can build consensus on goals and effects, but rather on the way they can navigate this consensus-building process at later stages of the partnering process.

This paper is largely conceptual and is based on a close reading of the existing partnering and evaluation literature. It was inspired by the practical observation that CSPs aiming for systemic change run the risk of becoming part of the problem instead of part of the solution—with a negative role to be played by the used ToC. We coupled this with an assessment that most of the partnering literature fails to consider systemic change and the ultimate impact of the CSP. This is partly due to methodological problems that are always attached to complex interventions. We also noted that the almost paradigmatic status of partnering for complex societal problems makes it difficult for donor organisations and supporters to embrace a ‘learning’ approach, without being able to deliver solid impact assessments. A systemic problem is then simplified to enable the partnership to define key performance indicators. There is considerable pressure to apply relatively instrumental ToCs for CSPs that have the intention to create systemic change. As a result, ToCs are rarely aligned with the complexity of the problem.

This contribution developed a Complexity-Sensitive approach to the use of ToCs. We defined four problem-oriented and four process-oriented components for the effective use of a ToC for CSPs aimed at varying degrees of complexity. We created a mapping construct based on societal triangulation to assess the complexity of problem, the complexity of the partnering configuration and the alignment complexity of linking these two. By developing these tools, we have reacted to calls from partnering practice and research to apply ToCs that are more complexity sensitive. However, each of these tools needs further validation. For example, we defined five-point scales, but further testing is needed to fine-tune these scales and the categories they represent.

This paper argues that specific combinations of problems and partnership configurations are more likely to be successful than others. The propositions—clustered around the four stages of partnering—define how we expect these alignment challenges to materialise. This paper also suggests that a new type of partnership research has emanated from combining partnership research with ToC development. More action research is needed, in which researchers engage in interventions themselves, not only to get longitudinal and dynamic results, but also to help further develop the partnership. More traditional forms of research (including quantitative research, control groups, randomised sampling) seem ill-equipped to cover dynamic and systemic change problems. Action research is not new, but is not yet well established in the field of cross-sector partnerships. If researchers are interested in enhancing the potential of partnerships for systemic change, this line of research seems particularly relevant and promising.

## Footnotes

1 Cf. https://systemicchange.wordpress.com/systemicchange [visited November 1 2017].

2 Note that some authors use the concepts of Programme Theory and Theory of Change interchangeably (Jackson 2013; Rogers 2008; Weiss 1995).

3 For a slightly different opinion, see Gray and Stites (2013) and Kolk (2013).
